# Delayed Tamponade after Traumatic Wound with Left Ventricular Compression

**DOI:** 10.1155/2016/2154748

**Published:** 2016-08-29

**Authors:** Fahad Almehmadi, Mark Chandy, Kim A. Connelly, Jeremy Edwards

**Affiliations:** ^1^Division of Cardiology, Department of Medicine, University of Toronto, Toronto, ON, Canada; ^2^King Saud Bin Abdulaziz University for Health Sciences, Jeddah, Saudi Arabia; ^3^Keenan Biomedical Research Centre, Li Ka Shing Knowledge Institute, St. Michael's Hospital, University of Toronto, Toronto, ON, Canada; ^4^Division of Cardiology, Terrence Donnelly Heart Centre, St. Michael's Hospital, University of Toronto, Toronto, ON, Canada

## Abstract

Delayed cardiac tamponade after a penetrating chest injury is a rare complication. The clinical diagnosis of tamponade is facilitated with imaging. We present a case report of a 23-year-old male who was brought to emergency after multiple stab wounds to the chest. After resuscitation and repair of laceration of right internal mammary artery and right ventricle, he was discharged but later returned with shortness of breath. Echocardiography revealed a rare case of delayed pericardial tamponade causing left ventricular collapse. The pericardial effusion was treated with emergent pericardiocentesis and later required a thoracoscopy guided pericardial window for definitive management.

## 1. Case Presentation

A 23-year-old male was brought to the emergency department after being stabbed 5 times in the chest and abdomen. He was resuscitated and an emergency chest tube was inserted for a left sided hemothorax. The Focused Assessment with Sonography in Trauma (FAST) was negative but later showed a progressively enlarging pericardial effusion. He was subsequently taken to the operating theater for a thoracic exploration.

After sternotomy, it was apparent that the knife transected the left internal mammary artery (LIMA) and lacerated the right ventricle (RV) just below the pulmonic valve level. The LIMA was ligated, RV was repaired, and a pericardial window was created. Exploration of the other wounds did not reveal any deeper penetrating injuries. His postoperative recovery was rapid and a subsequent echocardiogram showed no fluid recollection. He was discharged home shortly after.

**Figure 1:**
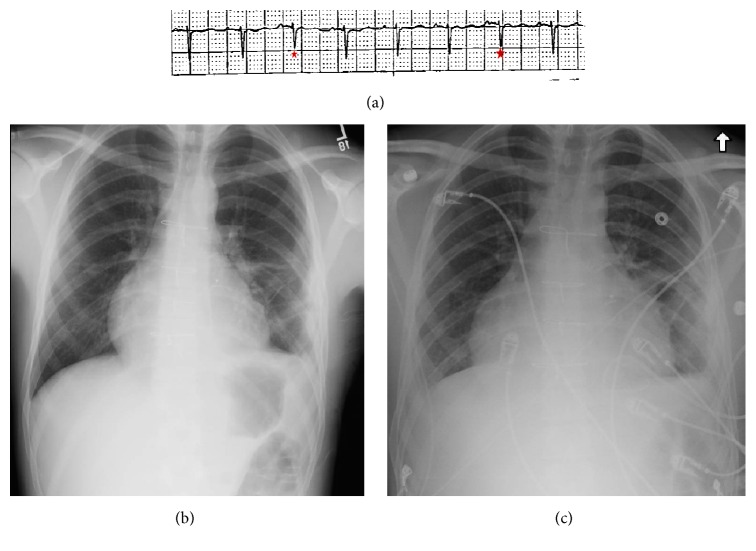
(a) Electrocardiographic strip showing sinus tachycardia and evidence of electrical alternans. (Note the smaller voltage of the QRS complexes annotated compared to others.) Posterolateral chest X-ray at last discharge (b) and at presentation (c) showing the increased size of cardiac silhouette.

Ten days later, he presented to the emergency department again but now complained of dyspnea, orthopnea, pleuritic chest pain, and lower limb edema. He showed typical clinical features of Beck's triad (i.e., hypotension, elevated JVP, and muffled heart sounds). He also had a significant pulsus paradoxus of 14 mmHg. The ECG showed electrical alternans (Figure 1), and a chest X-ray showed enlarged cardiac silhouette compared to chest X-ray before discharge (Figures 1(b) and 1(c)). A bedside echocardiogram performed in emergency department showed a new pericardial effusion with signs suggestive of tamponade physiology. Fluid resuscitation started, and he was urgently transferred to the Coronary Care Unit (CCU) for further care.

**Figure 2:**
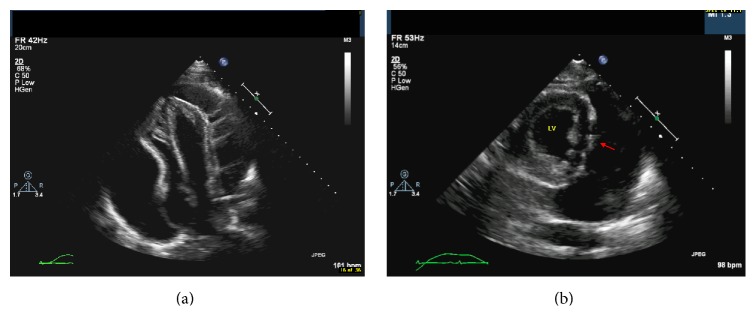
(a) Apical 4-chamber view on transthoracic echocardiogram showing large circumferential pericardial effusion with right atrial and ventricular compression. (b) Parasternal long-axis echocardiographic view of the left ventricle showing straightening of the LV lateral wall in diastole (red arrow).

An urgent bedside echo showed a large circumferential pericardial effusion with collapse of all cardiac chambers including a partial collapse of the left ventricle (Figures 2(a) and 2(b)) (Supplementary Videos A and B in Supplementary Material available online at http://dx.doi.org/10.1155/2016/2154748).

An emergency echo guided pericardiocentesis was performed but only 200 mL of bloody pericardial effusion was extracted; however, there was immediate improvement in his clinical status. A repeat transthoracic echocardiogram showed reduction in the effusion size with resolution of tamponade. The patient later underwent a thoracoscopy assisted pericardial window to drain the effusion. He is currently enjoying an active life one year after his discharge.

## 2. Discussion 

Penetrating cardiac trauma has a high mortality rate. While mortality has decreased with advances in emergency medical services [1], less than 10% will reach the hospital alive. Among the survivors that present to the emergency department, 70% will have evidence of acute tamponade. Of those that did not survive, 20% were found to have tamponade [2]. Thus, even if patients survived their initial trauma, patients are at risk of delayed cardiac tamponade.

Delayed cardiac tamponade is a well-described entity after both blunt [3] and penetrating trauma [1]. Pericardial effusion can be seen late in up to 22% of penetrating chest injuries [4] but luckily only a small fraction of these will present in clinical tamponade. Less than 50 cases of delayed pericardial effusion have been reported in literature.

A diagnostic and therapeutic challenge: delayed cardiac tamponade can occur up to 100 days after the index injury, and it may have an atypical presentation [5]. The etiology of the late presentation is unclear, although several mechanisms have been proposed including clot sealing a partial tear that either allows a slow leak into the pericardial sac or suddenly dislodges leading to more rapid accumulation [3]; autoimmune pericarditis and subsequent pericardial effusion; and unidentified laceration of a coronary artery with subsequent spasm that later hemorrhages [1].

Cardiac tamponade is a clinical diagnosis. Imaging modalities are vital in confirming clinical suspicion. Echocardiography continues to be the preferred imaging modality given its efficacy, availability, and ease of use. The presence of a pericardial effusion, chamber collapse, dilated inferior vena cava, and variation in mitral and tricuspid inflow are the hallmarks of pericardial tamponade on echocardiography. Right atrial (RA) systolic and right ventricle (RV) diastolic collapse has been described as an early sign of rising intrapericardial pressure, with the former being more sensitive and the latter being more specific [6]. Indeed, the longer duration of RA or RV collapse during a given cardiac cycle, the more severe the tamponade and the more specific the finding in echocardiography [7].

The collapse of left sided cardiac chambers is a more ominous sign. Left atrial collapse is more specific, but it is observed in only 25% of patients [6]. Left ventricular (LV) collapse in the setting of pericardial tamponade is very rare given its higher intracavitary pressure and the thicker wall. It has been observed in few cases of localized postoperative effusion and in circumferential pericardial effusion [8, 9]. Our case demonstrates an indentation of LV lateral wall in early diastole in parasternal short axis clips.

The treatment of delayed pericardial tamponade relies on resuscitation while preparing for urgent surgical evacuation. The optimal surgical strategy is unknown given the paucity of data that is mostly derived from case reports or case series. The standard of care for treatment is a subxiphoid pericardial window. Our patient did well with thoracoscopy guided pericardial window with a good clinical response and no reaccumulation of fluid. Sternotomy is most likely justified in case of hemodynamic instabilities despite technically successful pericardiocentesis.

## Supplementary Material

Echocardiography showing a large circumferential pericardial effusion with collapse of all cardiac chambers including a partial collapse of the left ventricle.
